# Factors Associated with Housing Stability Among Individuals with Co-Occurring Serious Mental Illness and Substance Use Disorders Receiving Assertive Community Treatment Services

**DOI:** 10.1007/s10597-024-01443-8

**Published:** 2024-12-30

**Authors:** Yeqing Yuan, Jennifer Manuel

**Affiliations:** 1 Department of Social Work, Samuel Merritt University, Oakland, CA USA; 2 University of Connecticut School of Social Work, CT Hartford, USA

**Keywords:** Housing stability, Assertive community treatment, Service engagement, Chart review, Co-occurring disorders

## Abstract

Assertive Community Treatment (ACT) is a community-based, multidisciplinary mental health treatment model with improved housing stability as a treatment goal. We know little about factors contributing to housing stability among ACT participants with co-occurring serious mental illness and substance use disorders, who account for 30% of the ACT participant population. Informed by the behavioral model of health service use, the present study aimed to examine the relationship between housing stability and theoretically relevant factors. We retrospectively abstracted the data from two ACT teams’ treatment service planning and tracking system. Stable housing was defined by living in a private residence or permanent supportive housing throughout the assessment periods; unstable housing was defined by having at least one unstable housing situation (e.g., jail or prison) throughout the assessment periods. The sample included 57 individuals with 272 assessment charts. Multivariate logistic regression results show that service engagement was associated with housing stability and that receiving housing supportive services was inversely associated with housing stability. The findings support prior literature in that service engagement remained a “critical ingredient” of the ACT model and highlights the importance of the supportive aspect of housing services in improving housing stability among individuals with co-occurring disorders.

## Introduction

An estimated 3.6 million adults in the United States have co-occurring serious mental illness (SMI) and substance use disorders (SUDs; henceforth referred to as CODs), comprising 27% of the entire population with SMI (Substance Abuse and Mental Health Services Administration, 2020). Compared to those with SMI only, individuals with CODs are more likely to experience housing instability such as homelessness, frequent residential relocation, and poor housing quality (Brunette et al., 2004; Drake et al., 1991). When experiencing homelessness and other forms of housing instability, individuals with CODs are more likely to experience worsened psychiatric symptoms and substance use, victimization, and social disconnection (Elbogen et al., 2023; Gabrielian et al., 2018). Housing instability, along with other adverse life events experienced by this population such as criminal justice involvement, unemployment, poverty, chronic illness, and marginalization, make individuals with CODs a particularly vulnerable and underserved group (Ahmad et al., 2020; Singh et al., 2019; Swope & Hernández, 2019). Currently, only a few interventions have shown improved housing outcomes among individuals with CODs, including Pathways Housing First (Tsemberis et al., 2012) and the integration of permanent supportive housing and Maintaining Independence and Sobriety Through Systems Integration, Outreach, and Networking (MISSION; Smelson et al., 2016; Smelson et al., 2018).

Assertive Community Treatment (ACT) is a widely implemented community-based mental health treatment model (Bond & Drake, 2015). It is an integral part of the Housing First model to reduce chronic homelessness and improve housing stability (Tsemberis & Eisenberg, 2000). Initially developed in the early 1970s for individuals with SMI (Stein & Test, 1980), ACT provides individualized community-based multidisciplinary services, including medical, psychosocial, and rehabilitation services. Key elements of the ACT model are assertive engagement, delivery of services in the community, high intensity of services, holistic and integrated services by multidisciplinary teams, and continuity of care (Bond & Drake, 2015).

Several studies demonstrated positive housing outcomes among individuals receiving ACT services. In a meta-analysis on the effectiveness of ACT among individuals experiencing homelessness and mental illness (Coldwell & Crane, 2007), participants in 8 of 10 studies had significantly reduced homelessness. As noted by the authors, the positive impact of ACT on housing stability is likely due to the key processes of ACT, which provide opportunity to successfully engage individuals with SMI and provide social supports necessary to access and maintain stable housing (Coldwell & Bender, 2007).

Despite the promising results, studies have largely focused on participants with mental illnesses alone (without substance use issues). A limited number of studies examined housing stability outcomes among ACT participants with CODs (Drake et al., 1998; Meisler et al., 1997; Morse et al., 2006), who account for 30% of the entire ACT consumer population. Findings from these studies showed promising yet inconsistent evidence on ACT’s effectiveness regarding housing stability. For example, in a clinical trial examining the treatment effects of integrated ACT (i.e., the same clinician provides both mental health and substance use treatment in a coordinated manner) versus ACT only on multiple outcomes among 149 individuals with CODs during 24 months, participants receiving either form of ACT had more days in stable housing than those in a control group (Morse et al., 2006). However, in another study examining the effects of ACT among 223 individuals with CODs during a 3-year period, the authors did not find positive effects of ACT on housing stability, defined as the number of days living in stable community residences (Drake et al., 1998). These findings suggest that additional knowledge is needed to understand the unique needs and challenges in improving housing stability among those with CODs in the existing ACT model. The present study aimed to examine factors associated with housing stability among individuals with CODs receiving ACT services.

## Theoretical Framework

The present study was guided by the behavioral model of health service use (Andersen, 1995). Originally developed in the 1960s to understand health service use among families, the model posits that individual’s use of services is a function of three types of factors: predisposing, enabling, and need factors (Andersen, 1995). Predisposing factors refer to the sociocultural characteristics of individuals that exist prior to their illness. Enabling factors refer to conditions that may be changed by individual and social efforts and that could enable access to health services. Need factors refer to perceived health care needs or objective health indicators. The behavioral model has been later revised to expand its outcome from health service use to health outcomes such as health status and consumer satisfaction. Because housing stability has been defined in the literature as the “ability to access housing that promotes individuals’ optimal health and quality of life over time” (Sylvestre et al., 2009), we consider it one form of health behaviors that is influenced by the multilevel factors outlined by the behavioral model. Acknowledging the model’s limitations in insufficiently addressing the structural context, a large body of research has used this framework to predict health and behavioral health service use (e.g., Babitsch et al., 2012; Koegel et al., 1999; Lemming & Calsyn, 2004; Roberts et al., 2018).

Limited research has examined factors associated with housing stability among individuals with CODs (Bebout et al., 1997; Tsai et al., 2010). In the broader literature concerning individuals with mental illnesses, the following factors have been found to be associated with housing stability.

### Predisposing Factors

Research has consistently found individuals who are younger (Lipton et al., 2000; Min et al., 2004; Pickett-Schenk et al., 2007; Tulloch et al., 2010; Wong et al., 2008), are male (Pearson et al., 2009; Pickett-Schenk et al., 2007; Pollio et al., 2000), or have a minority racial and ethnic background (Pickett-Schenk et al., 2007) are more likely to experience housing instability compared to their counterparts. In addition, having a sense of control and meaningful daily structures can also help people maintain housing stability (Crane & Warnes, 2007; Padgett, 2007).

### Enabling Factors

Having a positive relationship with family, friends, and service providers is positively associated with housing stability (Clarke et al., 2000; Goering et al., 1997). Accessibility and utilization of behavioral health services, including inpatient and outpatient mental health treatment (Caton et al., 1993; Lettner et al., 2016; Wong et al., 2008) and social support services such as case management services, vocational assistance, and housing assistance (Min et al., 2004; Pollio et al., 2000; Wong et al., 2008), were also found to be positively associated with housing stability. In addition, family disruption and stigma may also play a role in housing stability.

### Need Factors

Having a psychotic disorder (Lettner et al., 2016; Segal et al., 1992) and a history of homelessness (Lettner et al., 2016; Min et al., 2004; Wong et al., 2008) are both inversely associated with housing stability. In addition, substance use is a prominent barrier that has been consistently found to be inversely associated with housing stability (Caton et al., 1993; Lipton et al., 2000; Tulloch et al., 2010).

The behavioral model of health services use informed the selection of theoretically relevant factors influencing housing stability at each level in this study. Specifically,

the following hypotheses were tested:

1. Theoretically relevant predisposing factors, such as age, gender, and race and ethnicity, are significantly associated with housing stability.
2. Theoretically relevant enabling factors, such as treatment engagement and service use, are significantly associated with housing stability.
3. Theoretically relevant need factors, such as mental health diagnosis, substance use severity, and risk to self and others, are significantly associated with housing stability.

## Method

### Study Design

The present study is part of a mixed-methods study aiming to understand housing stability among individuals with CODs receiving ACT services. The present study aimed to quantitatively examine the relationship between housing stability and theoretically relevant factors. Data were retrospectively abstracted from two ACT teams in New York state: one in an urban setting and the other one in a suburban setting. The urban team primarily served a Black and Latino clientele population and operated as an independent ACT team, while the suburban team was part of a state psychiatric hospital. Both teams had psychiatrists, nurses, social workers, employment specialists, case managers, and peer specialists as required by the original ACT model (Stein & Test, 1980; Bond & Drake, 2015). Admission criteria for both teams included a diagnosis of serious mental illness (i.e., major depression disorder, bipolar disorder, schizophrenia, or schizoaffective).

Data were abstracted from each agency’s internal treatment service planning and tracking system. The system contains service assessment and planning data routinely collected by ACT service providers. Assessments are conducted upon participants’ admission to ACT and every 6 months thereafter. The database captures six domains: (a) demographic characteristics (e.g., age, gender, race and ethnicity, education, income, insurance, living situation, criminal justice involvement); (b) clinical information (e.g., mental health diagnosis, medical problems, medication adherence); (c) service utilization (e.g., medical treatment, psychiatric inpatient, emergency room use); (d) well-being (e.g., risk behavior, substance use); (e) treatment plan (e.g., current service plan, engagement level); and (f) functioning (e.g., level of current assistance).

Ethical approval has been granted by the New York University Institutional Review Board and both participating agencies.

### Sampling

The sampling frame is a list of ACT teams provided in the New York State Office of Mental Health’s ACT Interactive Data Reports, an online tableau visualization tool that allows the public to view and interact with ACT data. The authors identified and invited 23 teams in which at least 20% of clients are individuals with CODs, and two ACT teams confirmed participation. In each team, all current clients with a diagnosis of substance use disorder were sampled. The diagnosis of substance use disorders was made *a priori* by the ACT staff during clinical assessments based on the *Diagnostic and Statistical Manual of Mental Disorders* (5th ed.; American Psychiatric Association, 2013).

At the individual level, assessment charts were collected for the prior 3 years for each eligible ACT participant. For example, if a client had been receiving ACT services for longer than 3 years, charts for the last 3 years and the admission chart were abstracted (a total of seven charts); if the client had been receiving ACT services for less than 3 years, all charts were abstracted. Because individuals had varying lengths of stay, the number of charts varied by individual, resulting in an unbalanced clustered data structure.

### Procedures

ACT staff members identified eligible participants, obtained eligible charts, and redacted clients’ personal health information before providing the data to the author. Consent was not necessary for this study because we only used de-identified data.

Data abstraction was performed by the author and a trained research assistant between January and March 2021. Following literature suggestions (Vassar & Matthew, 2013), several strategies were used to improve the validity of the abstraction, including formulating a priori research questions before performing the chart review, using a standardized abstraction form, creating a procedural manual, and training and monitoring the additional data abstractor. All participants were receiving ACT services at the time of data abstraction.

### Measures

All measures were assessed by ACT staff members based on their clinical observation or interactions with clients. The primary dependent variable was a categorical variable of housing status during the past 6 months with the following five mutually exclusive response options: (a) private residence (e.g., apartment, house, dorm, rented room, housing choice voucher); (b) housing with supports (e.g., permanent supportive housing such as scattered-site supportive housing); (b) temporary supportive housing (e.g., Office of Mental Health-licensed housing, crisis residence, halfway housing, HIV/AIDS housing); (c) in an institution (e.g., hospital, jail or prison, residential treatment facility); (d) unstable housing (e.g., couch surfing, living with a friend short term, living with family members, imminent eviction); and (e) homelessness (e.g., homeless shelter, drop-in shelter, on the street, squatting, hotel via Department of Social Services placement). For the current analysis, the five categories were collapsed into a binary variable of stable or unstable housing, with stable housing including private residence and supportive housing, and unstable housing including institutional housing, unstable housing, and homelessness.

Informed by Andersen’ behavioral model of health service use (1995), we included the following theoretically relevant independent variables. For predisposing factors, we included age, gender, and race and ethnicity (e.g., non-Hispanic White, non-Hispanic Black, Hispanic, other). For enabling factors, we included length of stay, which was a continuous variable operationalized by calculating the number of years between ACT admission and the date of data abstraction (January 1, 2021); housing service, which was a binary variable indicating whether the participant received housing and housing-related supportive services as part of their current service plan; and employment, which was a binary variable indicating whether the participant was currently employed in any form. It also included engagement, which had four mutually exclusive categories: (a) well engaged (actively involved in working on life goals with staff, having a strong connection with all or most ACT team members); (b) somewhat engaged (takes some initiative communicating needs or is beginning to take a role in development of life goals with staff); (c) minimally engaged (requires significant outreach or minimally connected to at least one staff member); and (d) not engaged at all (does not respond to outreach, no communication with staff). For the current analysis, we operationalized engagement as a binary variable with two mutually exclusive groups: engaged (well engaged and somewhat engaged) and disengaged (minimally engaged and not engaged).

For need factors, we included mental health diagnosis, which was assessed by ACT staff members based on the *Diagnostic and Statistical Manual of Mental Disorders* (5th ed.; American Psychiatric Association, 2013) at admission. The current analysis operationalized the variable as a categorical variable with three mutually exclusive categories: schizophrenia, schizoaffective, and bipolar or depression. Medical problem was operationalized as a binary variable indicating whether the participant had any current medical problem. Mandated was a binary variable indicating whether the participant was mandated to receive ACT treatment as part of court-ordered assisted outpatient living.

Intensive service use was a binary variable indicating whether the participant had used any of the following services in the past 6 months: (a) psychiatric emergency room psychiatric hospitalization or (b) emergency room or hospitalization due to general medical concerns. Substance use was measured by a question determining whether the participant had used any of 14 substances in the past 6 months. For the current analysis, we operationalized it as (1) a continuous variable indicating the cumulative count of substance types used during the 6 months prior to assessment and (2) a categorical variable with six types of substances: alcohol, marijuana, tobacco, cocaine/crack cocaine, opioids, and other drugs. In addition, we included risk behaviors, measured with a question determining whether the participant engaged in any of 14 risk behaviors in the past 6 months, including expressed suicidal ideation, expressed homicidal ideation, and damaged or destroyed property. For the current analysis, this was operationalized as a continuous variable indicating the cumulative count of risk behavior types engaged in during the 6 months prior to assessment.

### Analysis

First, proportions and means were calculated for participant characteristics at ACT admission to present baseline sample characteristics. Next, differences in sample characteristics were examined by housing status. Different units of analysis were used depending on whether the predictor was time varying. For time-invariant predictors (i.e., age, gender, race and ethnicity, mental health diagnosis, and length of stay), the analysis compared person-level characteristics by housing status. Unstable housing referred to an individual ACT client experiencing at least one unstable housing period during all assessment periods, which ranged between six months to three years depending on participants’ program entry date. Fisher’s exact tests (for dichotomous variables) or point biserial correlation (for continuous variables) were conducted to examine the association between housing status and each time-invariant predictor.

For time-varying predictors (i.e., employment, medical problems, intensive service use, number of substance use, type of substance use, risk behaviors, service engagement, mandated, housing service use), the analysis compared chart-level characteristics by housing status. Unstable housing refers to any service assessment period during which an unstable housing situation had occurred. Unadjusted logistic regression models were computed to estimate the probability of having an unstable housing period as a function of each time-varying predictor.

Finally, multivariate logistic regression models were computed to examine the association between housing status and the hypothesized predisposing, need, and enabling variables for the pooled sample. Besides the theoretically relevant independent variables previously described, the multivariate models included an indicator term for time and adjusted standard errors using the vce(cluster) command in Stata 17 to account for multiple observations for the same individuals (Wooldridge, 2003). Exponentiated coefficients were generated that can be interpreted as odds ratios. Missing data accounted for less than 5% of the total data and were omitted.

## Results

The overall sample consisted of 57 individuals with a total of 272 assessment charts. Six individuals had one assessment, eight had two assessments, three had three assessments, eight had four assessments, four had five assessments, seven had six assessments, and 21 had all seven assessments. Fifteen of the 57 individuals experienced unstable housing during at least one assessment period (26.3%), and the remaining individuals had stable housing throughout the reviewed assessment periods, represented by 34 charts with unstable housing status of the 272 reviewed charts.

### Baseline Sample Characteristics

The first two columns in Table 1 show participant characteristics upon entering ACT. The sample had a mean age of 39.9 (*SD* = 12). Consistent with the overall ACT client population (Office of Mental Health, 2022), women accounted for 14.3% of the sample. A substantial portion of the sample was non-Hispanic Black and Hispanic (37.5% each). More than half the participants had a diagnosis of schizophrenia (50.9%), had at least one medical problem (56.1%), were mandated to receive ACT services (56.1%), and were receiving housing services (52.6%). At the time of data extraction, most of the sample had received ACT services for between 1 and 3 years, and two participants had received ACT services for longer than 10 years.

**Table 1:** Participant characteristics by Housing Status

	Baseline characteristics		Stable housing			Unstable housing			Fisher’s exact test^c^		
	*n* or *M*	% or *SD*	*n* or *M*		% or *SD*	*n* or *M*		% or *SD*			
*Time-invariant predictors* (*n = 57*)											
Individual			42		73.7	15		26.3			
Age^a^	39.9	12.0	38.9		11.7	42.7		12.9	0.14		0.31
Female	8	14.3	7		17.0	1		6.7	0.43		
Race and ethnicity									1		
Non-Hispanic White	8	14.3	6		14.6	2		13.3			
Non-Hispanic Black	21	37.5	15		36.6	6		40			
Hispanic	21	37.5	15		36.6	6		40			
Other	6	10.7	5		12.2	1		6.7			
Mental health diagnosis									0.79		
Schizophrenia	29	50.9	21		50.0	8		53.3			
Schizoaffective	16	28.1	11		26.2	5		33.3			
Bipolar or depression	12	21.1	10		23.8	2		13.3			
Length of stay^b^									0.69		
< 1 year	5	10.4	3		8.6	2		15.4			
1–3 years	23	47.9	18		51.4	5		38.5			
3–10 years	18	37.5	12		34.3	6		46.2			
> 10 years	2	4.2	2		5.7	0		0			
Continuous^a^	3.8	3.6	3.9		3.9	3.5		2.8	− 0.04		0.77
*Time-varying predictors* (*n = 272*)									*OR*		95% CI
Charts			238		87.5	34		17.5			
Employed	5	9.1	31		13.2	4		11.8	0.88		[0.29, 2.67]
Have medical problems	32	56.1	93		39.1	12		35.3	0.85		[0.40, 1.80]
Used intensive service	44	80.0	115		48.7	21		63.6	1.84		[0.87, 3.91]
# Substance used in past 6 months^a.d^	2.0	1.5	1.59		1.69	1.9		2.0	1.11		[0.92, 1.35]
Substance used in past 6 months^a^											
Alcohol	22	38.6	60		25.2	12		35.3	1.62		[0.76, 3.47]
Marijuana	34	59.7	120		50.4	15		44.1	0.78		[0.38, 1.60]
Cocaine/Crack Cocaine	16	28.1	39		16.4	11		32.4	2.44		[1.10, 5.41]
Opioids	7	12.3	21		8.8	5		14.7	1.78		[0.62, 5.09]
Tobacco	18	31.6	79		33.2	8		23.5	0.62		[0.27, 1.43]
Other drugs	10	17.5	26		10.9	8		23.5	2.51		[1.03, 6.11]
Risk behaviors in past 6 months^a^	1.2	1.6	0.65		1.41	0.8		1.34	1.07		[0.84, 1.36]
Engagement	35	62.5	190		80.2	15		44.1	0.20		[0.09, 0.41]
Mandate	32	56.1	105		44.1	20		58.8	1.81		[0.87, 3.75]
Housing services	30	52.6	120		50.4	29		85.3	5.70		[2.14, 15.23]

^a^ Point biserial correlation results are shown instead of Fisher’s exact test

^b^ Length of stay until the date of data extraction (January 1, 2021)

^c^ Point biserial correlation was performed for continuous variables (i.e., age and a continuous variable of length of stay). Correlation co-efficient (left) and p value (right) are shown. Otherwise, the p-value for fisher’s exact test is shown

^d^ Not mutually exclusive due to polysubstance use

At the time of admission, only five individuals were employed either full time or part time. During the 6 months before admission, most of the sample (80%) used intensive care, including emergency room and psychiatric hospitalization. The most frequently used substance was marijuana (*n* = 34, 59.7%), followed by alcohol (*n* = 22, 38.6%) and tobacco (*n* = 18, 31.6*%).* The mean number of kinds of substances used in the sample was two (*SD* = 1.5), and the mean number of kinds of risky behaviors was 1.2 (*SD* = 1.6). A sizeable segment of the sample (62.5%) was engaged in ACT services at the time of admission.

### Sample Characteristics by Housing Status

Depending on whether a predictor was time varying or time invariant, person-level and chart-level sample characteristics are presented by housing status (Table 1, Columns 4–9). Although not statistically significant, participants with unstable housing experiences were more likely to be older, male, either Non-Hispanic Black or Hispanic, carrying a primary diagnosis of either schizophrenia or schizoaffective, and have been receiving ACT for 3 to 10 years. At the chart level, unstable housing periods were more likely to happen when a participant was unemployed, had no medical problems, was receiving intensive services, was using more kinds of substances, especially alcohol, cocaine/crack cocaine, opioids, and other drugs, was engaging in fewer risky behaviors, was less engaged in ACT services, was mandated to receive ACT services, and was receiving housing-related services. Significant bivariate relationships were found between housing status and a number of predictors, including service engagement, cocaine/crack cocaine use, and use of other drugs.

### Multivariate Analysis of Factors Associated with Unstable Housing Status

Table 2 presents the multivariate relationships between unstable housing status and theoretically relevant factors after controlling for time and adjusting for robust standard errors. The adjusted model shows that in any assessment period, theoretically relevant enabling factors, including treatment engagement and service use, were significantly associated with housing stability. Specifically, those engaged in ACT services were less likely to experience unstable housing than those disengaged from ACT services (AOR = 0.14, 95% CI [0.03, 0.67]), when holding constant all other variables. Those receiving housing services were more likely to be experiencing unstable housing than those without housing services (adjusted odds ratio [AOR] = 6.49, 95% CI [1.45, 29.06]), when holding constant all other variables. In contrast, theoretically relevant predisposing factors (i.e., age, gender, and race and ethnicity) and theoretically relevant need factors (i.e., mental health diagnosis, substance use severity, and risk to self and others) were not significantly associated with housing stability.

**Table 2:** Multivariate relationships between Housing Stability and theoretically relevant factors

	AOR	Robust SE	95% CI	*P* Value
Predisposing factors				
Age	1.05	0.03	[1.00, 1.11]	0.06
Female	0.18	0.23	[0.01, 2.21]	0.18
Race and ethnicity^a^				
Non-Hispanic Black	0.37	0.41	[0.04, 3.19]	0.37
Hispanic	0.43	0.36	[0.08, 2.19]	0.31
Other	0.11	0.12	[0.01, 0.94]	0.04
*Need factors*				
Diagnosis^b^				
Schizoaffective	3.36	3.08	[0.56, 20.26]	0.19
Bipolar or depression	0.42	0.32	[0.09, 1.91]	0.26
Medical problems	0.35	0.27	[0.08, 1.57]	0.17
Mandate	1.79	1.04	[0.58, 5.57]	0.31
Substance use	1.01	0.13	[0.78, 1.31]	0.92
Risk behaviors	0.88	0.18	[0.59, 1.31]	0.52
Intensive service	2.61	1.82	[0.67, 10.22]	0.17
*Enabling factors*				
Length of stay	0.87	0.11	[0.69, 1.10]	0.25
Housing services	6.49	4.96	[1.45, 29.06]	0.01
Engagement	0.14	0.11	[0.03, 0.67]	0.01
Employment	0.81	0.59	[0.19, 3.41]	0.77

^a^Reference category: non-Hispanic White

^b^Reference category: schizophrenia

## Discussion

Using service assessment and planning records from two ACT teams, the present study examined factors associated with housing stability among individuals with CODs receiving ACT services. Consistent with our hypothesis that theoretically enabling factors are associated with housing stability, our key finding suggests that individuals who are engaged with ACT services are more likely to be living in stable housing situations. Our findings did not support other hypotheses that theoretically predisposing and needs factors are associated with housing stability. Our study is one of the first studies confirming the importance of service engagement among those with CODs. This finding supports and complements prior literature indicating that service engagement remains a “critical ingredient” of the ACT model (Manuel et al., 2013; Stanhope, 2012). It also highlights the importance of ACT services as part of the original Housing First model in improving and sustaining housing stability. Although research and policies have largely emphasized creating permanent and affordable housing as a pathway to eliminating homelessness, the current findings call for increased attentions on importance of increased engagement with supportive service in improving housing stability among individuals with CODs.

Service engagement may improve housing stability through several pathways. First, as documented extensively in the literature (Dixon et al., 2016; Lindsey et al., 2019; Schley et al., 2012), service engagement is directly linked to positive behavioral health outcomes, which in turn promote housing stability by improving individuals’ capability of managing risky behaviors and personal responsibilities and reducing disruptive residential relocation due to institutionalization (e.g., incarceration, hospitalization). Second, as Stanhope (2012) explained, service engagement as a social process contributes to program effectiveness, especially in an intensive treatment setting like ACT where clients and service providers are expected to spend a considerable amount of time. The engagement process between clients and service providers helps personalize the housing experience and keeps individuals connected and supported, which are important components of subjective housing stability, as identified by Yuan and colleagues (2023). Furthermore, better engagement with staff members makes it possible for staff to “endorse” individuals during the process of housing placements.

The findings also suggest that individuals with CODs who are receiving housing-related supportive services are more likely to be in unstable housing situations. This may indicate that ACT teams are responsive to clients’ housing needs and allocating their services to those with the greatest needs. Similarly, the findings show that unstable housing is also more common when individuals receive more frequent intensive services, are mandated to receive ACT services, and have psychotic symptoms. This is consistent with prior literature showing that housing stability is inversely associated with intensive treatment services (Kerman et al., 2018; Yuan & Manuel, 2018). Consistent with the literature (Grinman et al., 2010; Tsai et al., 2014), the current study suggests that severe substance use, as evidenced by more types of substances used in the past 6 months, is more common among people with unstable housing episodes. Surprisingly, unstable housing was more common among those with no medical problems, whereas risk behaviors were more common among those in stable housing situations. These findings may have to do with measurement errors, because medical needs and risk behaviors are more frequently assessed and reported by staff members when a person has a stable housing situation. It could also be that the occurrence of serious medical conditions led to participants’ connection and engagement with ACT services, whereas those without medical problems were less interested in or in need of high intensity services such as ACT.

This study had several limitations that are worth noting. First, due to challenges accessing administrative data, the study used a small sample that was likely underpowered to detect significant relationships among the various predictors. The small sample size also limited the ability to examine the longitudinal relationships controlling for unobserved with group variations. In addition, the indicator for housing stability was limited to a binary variable. The present study could not assess the quality of housing or subjective experiences of participants’ living experience, which may provide important implications for service planning.

Despite these limitations, this study offers important implications for behavioral health service practices. First, given that housing stability is a primary service outcome for ACT and that ACT is transitioning to a time-limited service model (Huz et al., 2017), comprehensive assessments of housing needs and goals should be conducted early to facilitate early engagement regarding housing goals. Second, it is critical to practice proactive, continuous, and flexible engagement strategies, especially for clients with diverse racial and ethnic backgrounds and more demanding behavioral health needs. Because effectiveness is informed by both evidence-based interventions (Manuel et al., 2013) and practice wisdom from service providers, it is important to allocate resources and training for staff members to share resources and support to promote effective engagement efforts. Because substance use and high-intensity service users may still experience housing instability, it is important to examine organizational barriers such as stigma and practice competence rather than client-level barriers. Organizational leaders may facilitate engagement by providing training on knowledge and skills related to working with diverse clientele with substance use needs. Finally, other behavioral health services beyond ACT, such as intensive case management, might consider adopting successful ingredients from ACT, such as incorporating peer support, to improve housing stability in their client population.

This study also offers several important implications for future research. Although engagement has been studied extensively in the behavioral health services field, there is less clarity on service engagement with regard to housing outcomes, warranting an in-depth understanding of the “what” and “how” regarding engagement related to housing stability. Future research should document the need for service engagement, client preferences regarding engagement approach, the mechanisms through which engagement influences various housing outcomes, and programmatic and policy barriers to service engagement.
